# Depression, Contraception, and Ambivalence Concerning Fertility

**DOI:** 10.1007/s10508-024-02879-5

**Published:** 2024-06-03

**Authors:** Sina Kremer, Alexander L. Gerlach, Doris Erbe

**Affiliations:** Department of Psychology, University of Cologne, Pohligstr. 1, 50969 Cologne, Germany

**Keywords:** Contraceptive behavior, Depression, Ambivalence, Desire for children

## Abstract

Individuals suffering from depression exhibit a higher rate of unintended pregnancies, which are associated with negative outcomes for both parents and children. Often, unintended pregnancies result from contraceptive mistakes. Here, we examine the relationship between depression and the consistency of contraceptive behavior, testing ambivalence as a possible mediator. The analyses were based on cross-sectional data from the second and third waves of the German Relationship and Family Panel Pairfam. A German-speaking sample without children (*N* = 190; 117 female, 73 male), who reported not attempting to conceive or become pregnant during the last 12 months, was analyzed in comparison with a propensity score matched sample. Ambivalence was operationalized as the difference between the ideal and realistic number of children in wave 2. Data from wave 3 were used to assess contraceptive behavior. Depressed mood in wave 2 and consistency of contraceptive behavior in wave 3 were negatively correlated. After including ambivalence in wave 2 as a mediator in the model, the direct path between depressed mood and consistency of contraceptive behavior remained significant, with no significant mediation found. For men only, we observed a significant negative association of ambivalence with the consistency of contraceptive behavior in the last 3 months. No significant relationship was found between depressed mood and ambivalence. We conclude that future research aiming to better understand the consistency of contraceptive behavior should incorporate measures of ambivalence.

## Introduction

In Germany, one of three pregnancies is unintended. Overall, 46.9% of all German women who have ever been pregnant have been unintentionally pregnant at some point in their lives (Helfferich et al., 2016). It is important to note that the rates of unintended pregnancies are particularly high among people with depression (James-Hawkins et al., 2014; Maxson & Miranda, 2011; Wellings et al., 2013).

Research on unintended pregnancies is relevant because they are associated with negative outcomes for both parents and children (Cheng et al., 2009; Cruz-Bendezú et al., 2020; Herd et al., 2016; Korenman et al., 2002; Mohllajee et al., 2007). Children show generally poorer health status both physically and psychologically (Bosanac et al., 2003). Also, women with a history of depression who become pregnant unintentionally are at increased risk for developing prenatal and postpartum depression (Abajobir et al., 2016; Ferrari et al., 2021).

Unintended pregnancies are often a result of improper contraception. In the USA, contraceptive nonuse and inconsistent use account for 54% and 41% of unintended pregnancies, respectively (Sonfield et al., 2014). Accordingly, people with depression are often inconsistent in their use of contraception (Adan Sanchez et al., 2019; Callegari et al., 2015; Casola et al., 2017; Hall et al., 2012, 2013; Kosunen et al., 2003).

### Contraceptive Behavior in People with Depression

Several studies demonstrate increased rates of unintended pregnancies in people suffering from depression. In a meta-analysis, DiMatteo et al. (2000) examined depression as a risk factor for nonadherence to medical treatment in general. Individuals with depression exhibited a threefold risk of nonadherence compared to healthy individuals. Arguably, this risk can be transferred to the (in-)consistent use of contraceptive measures. Indeed, people with depression often do not use contraception at all or use it less consistently (Hall et al., 2012, 2013; Kosunen et al., 2003; Lehrer et al., 2006).

Little is known about how depression may impact on use and choice of contraceptive methods (Catalao et al., 2023). Since the consequences of unintended pregnancies are particularly negative for people with depressive disorders, it is important to better understand this relationship.

### Ambivalence in Fertility Desires and Fertility Expectations Among People with Depression

Various concepts in family planning exist in relation to fertility (Kuhnt & Buhr, 2016), including concepts like fertility desires, intentions, or expectations (Bühler, 2012; Miller, 2011). Concerning the measurement of reproductive preferences and desires, it has been argued that questions which force respondents to name a single number of desired family size may give the impression of certain or unambiguous reproductive goals that do not exist (Bühler, 2012). As such, studies like pairfam address desired family sizes under different criteria, i.e., how many children respondents would like to have based on their personal ideals, biographical experiences and expected living conditions, or perceived societal expectations and use a hybrid concept of fertility desires and intentions (Bühler, 2012; Kuhnt & Buhr, 2016; Suckow et al., 2009). Ambivalent reproductive desires, which are contradictory or not clearly established, are common, especially in young adults (Higgins et al., 2012). Pregnancy ambivalence, however, is defined and measured differently in different studies. For instance, some studies attribute pregnancy ambivalence as the midpoint of a bipolar scale on favorable vs. unfavorable attitudes toward getting pregnant, while other studies attributed ambivalence to persons who said they did not know or were unsure in response to a question about attitudes toward pregnancy (Miller et al., 2013).

In a number of studies (Crosby et al., 2002; Frost et al., 2007; Jaccard et al., 2003), ambivalence is attributed to individuals who scored positive or neutral on the attitudes toward pregnancy scale even though their situation was unfavorable for pregnancy, for instance, because of being underage. This concept has similarities to the concept of pairfam concerning the ideal vs. realistic desire for children, which will be explained later. Miller et al. (2014) defined ambivalence as different individual strong positive and negative feelings about a person, object, or goal that are simultaneously present (Miller et al., 2014). Interestingly, despite the different operationalizations of ambivalence, the association of ambivalence and inconsistent contraceptive behavior has been found consistently. Zabin et al. (1993) were the first to examine ambivalence in relation to pregnancy motivation and found that ambivalence was related to not using effective contraception (Zabin et al., 1993). Similarly, Miller et al. (2014) found that ambivalence influenced the use of condoms for contraceptive purposes negatively. Also, pregnancy ambivalence in young sexually active men was associated with a significantly lower likelihood of using contraception (Higgins et al., 2012).

Remarkably, Patel et al. (2015) not only found an increased risk of not having used contraception during the last intercourse among women with increased pregnancy ambivalence, but that depression was also a significant predictor of ambivalent pregnancy intentions.

When considering fertility expectations of people with mental disorders, it should be noted that planned behavior, such as that of family planning, is usually impaired by the fact that the course of mental disorders is difficult to predict (Krumm & Becker, 2006). A worsening of said conditions usually has far-reaching consequences that affect not only the individual but also the partnership, the professional situation and the general quality of life (Becker & Krumm, 2006).

Finally, the cognitive model of depression suggests generalized expectations of negative outcomes in afflicted individuals (Beck & Alford, 2009). Arguably, these negative expectations may also apply to fertility expectations. It can be assumed that fertility expectations, independent of desire for children, are also interpreted negatively and are characterized by a negative prediction in terms of failure and non-achievement of personal goals. While fertility desires are represented in the desired (ideal) number of children independently of the current situation and future obstacles, fertility expectations incorporate such negative expectations. Accordingly, people with depression will likely report a lower “realistic” number of children giving their life circumstances. The size of this discrepancy in turn may lead to a more ambivalent state torn between fertility desires and expectations. Depression has been shown to be associated with ambivalence (Deighton & Traue, 2006; Emmons & King, 1988) and with ambivalence about pregnancy in specific (Francis et al., 2015). Ambivalence concerning pregnancy avoidance may then in turn be related to contraceptive behavior.

While many studies have shown that consistency in contraceptive behavior is associated with ambivalence and/or depression and several studies demonstrate increased rates of unintended pregnancies in people suffering from depression, there is—to our knowledge—no study to date that examines the three variables ambivalence, contraceptive behavior, and depression in their reciprocal relationship and, in particular, examines the mediating role of ambivalence with respect to the relationship between depression and contraceptive behavior. As such, the assumed negative relationship between depression and consistent contraceptive behavior is assumed to be partially mediated by ambivalence.

### Aim of the Investigation

The aim of the present study was to examine the link between inconsistent contraception and depression. It was hypothesized that higher scores of depressive symptoms would be associated with lower consistency of contraception in the next wave (Hypothesis 1) and with higher ambivalence scores (Hypothesis 2). In addition, higher ambivalence scores were hypothesized to be associated with less consistent contraception in the next wave (Hypothesis 3). Finally, ambivalence was hypothesized to partially mediate the association between higher scores of depressive symptoms in wave 2 and lower consistency of contraceptive behavior in wave 3 (Hypothesis 4).

Analyses were based on data from the second and third wave of the German Family Panel pairfam (Panel Analysis of Intimate Relationships and Family Dynamics) release 11.0 (Brüderl et al., 2020; Huinink et al., 2011).^1^ For a detailed description of the study, see Huinink et al. (2011). The pairfam project is conducted as a collaboration between the Universities of Bremen, Chemnitz, Mannheim, and the Ludwig Maximilian University of Munich.

The observational study pairfam is an interdisciplinary panel study with a multi-actor design to investigate partnership and family living arrangements in Germany. The multi-actor design includes the independent interviewing of anchor persons as well as their partners, parents, and children. In addition to the multi-actor design, a multi-cohort design is also implemented, in which the anchor persons were randomly selected from the population of German-speaking persons of three birth cohorts. The survey started in 2008–2009 with a nationwide random selection of more than 12,000 anchor respondents. The face-to-face interviews were conducted by Kantar Public (formerly: TNS Infratest Sozialforschung). The pairfam study consists of a multi-topic survey in which the interactions and dynamics of the topics of partnership, parenthood and fertility, intergenerational relationships, and education and child development can be queried and analyzed.

## Method

### Participants

The target population for the pairfam study included all German-speaking individuals, regardless of nationality, living in private households in Germany. Subjects from three cohorts were recruited. The three cohorts consist of subjects born in the years 1991 to 1993, 1981 to 1983, and 1971 to 1973. In this study, we use the sample from the second wave, which was surveyed in 2009/2010, and included data of the third wave, which was surveyed in 2010/2011. The second wave was the first wave in which the presence of depression was assessed via the State-Trait Depression Scale (trait version; STDS-T). Consistency of contraceptive behavior was assessed as of wave 3 in order to realize a longitudinal model. Subjects were included if they had answered all questionnaires relevant to the study, did not have children (yet) in wave 3 and reported in wave 3 not having tried to conceive a child or get pregnant during the last 12 months but having been sexually active in the past 3 months. Also, we matched significantly depressed subjects with statistically similar non-depressed subjects using propensity score matching (see below). Analyses were based on the final sample of 190 (95 likely depressed and 95 not depressed). The average age in wave 2 was 23.81 years (SD = 6.36). 6.3% were married, 92.1% had never been married, and 1.6% were divorced. The sample consisted of 117 women and 73 men. Concerning the highest level of education, 4.2% of subjects reported not having any school leaving qualification, 9.5% reported having a lower secondary school leaving certificate, 32% reported having an intermediate secondary school leaving certificate, 6.8% reported having an advanced technical college entrance qualification, and 22.6% reported having a general university entrance qualification. 23.7% reported being currently enrolled.

### Measures

#### State-Trait Depression Scale-Trait (STDS-T)

The STDS-T of (Spielberger & Reheiser, 2009) was used to assess trait depressivity with the subscales Dysthymia (T-Dys, depression-positive items) and Euthymia (T-Eut, depression-negative items). The STDS-T is able to detect the presence of depression in samples from the general population (Krohne et al., 2002). The internal consistency of the total scale and the subscales is high with a Cronbach's alpha of 0.81–0.85 (Bortz & Schuster, 2016). Lehr et al. (2008) suggested that the optimal cutoff value for diagnosing depressive disorder on the STDS-T is at 25 points.

#### Ambivalence

In the present study, ambivalence was measured via the difference of two items, (“item *frt5*: "If you disregard all obstacles for once: Ideally, how many children would you like to have in total?" and item *frt6*: "Once you think realistically about children: how many children do you think you will have?" Response options were 7 = "no (additional) child" (7 was recoded to 0 for analysis); 1 = "one (additional) child," 2 = "two (additional) children," 3 = "three (additional) children," 4 = "four (additional) children or more."). An ambivalence value was calculated by subtracting the realistic number of children from the ideal number of children. Only child count statements from zero to four in frt5 were included. As such, if respondents indicated expecting less children then they would ideally like to have, the ambivalence value was positive. Higher values indicate higher ambivalence. If respondents expected more children then they would ideally like to have in total, the ambivalence score will be negative indicating a stronger antenatal position. Since values other than zero—both negative and positive values—are, on the other hand, hypothesized to be associated with higher scores in STDS, respondents with negative ambivalence scores were not included in the mediations analysis.

#### Contraceptive Behavior

Contraceptive behavior was measured via two items in wave 3 (item *sex5*: "Have you used contraception in the past 3 months?" and item *sex7*: "Please think about the last 3 months: How consistently did you use contraception?"). The latter question was asked only if subjects were heterosexual, had used contraception in the past 3 months, were not sterilized, and whose partner was not sterilized. Anchors entered their response on a five-point Likert-type scale (1 = "very inconsistent" to 5 = "very consistent"). Responses were then recoded to 2 = "very inconsistent" to 6 = "very consistent." Subsequently, both variables were combined into the variable consistency of contraceptive behavior (item *sex8*) on a six-point Likert-type scale (1 = "very inconsistent" to 6 = "very consistent"). This scale was based on the assumption that no contraception in the last 3 months (sex5 = 1) in sexually active subjects not aiming at getting pregnant or fathering children can be understood as the most inconsistent pole in consistency of contraception during the last 3 months.

### Statistical Analysis

For the mediation analysis, model 4 of the PROCESS macro (version 3.5) by Hayes (2017) was used within SPSS (version 29). This macro uses simple linear least square regressions to conduct the mediation analysis to determine unstandardized path coefficients of the total, direct, and indirect effects and bootstrapping with 10,000 iterations to estimate mediation effects using confidence intervals. To calculate the confidence intervals, we used heteroscedasticity consistent standard errors (Davidson & MacKinnon, 1993). Accordingly, effects were considered significant if the confidence interval did not include zero. The STDS-T sum score (*depr*) was the independent variable, ambivalence (*ambi*) represented the mediator, and contraceptive behavior (*sex22*) was the dependent variable. Gender, age, and relationship status were control variables. A mediation analysis was calculated including only subjects without children and propensity score matching was performed.

#### Propensity Score Matching

Due to the small proportion of subjects likely being clinically depressed (*N* = 95) as identified by using the cutoff value of the STDS-T (*x* > 24), propensity score matching was performed using R statistical software (version 3.6.3) to identify a healthy control group. The propensity score indicates the probability of treatment assignment as a function of the observed covariates. The propensity score makes it possible to design and analyze an observational (non-randomized) study in such a way that it mimics some of the special characteristics of a randomized controlled study (Austin, 2011). For the present study, subjects were matched with respect to gender, age, school graduation, and relationship status using nearest neighbor matching method. Nearest neighbor matching selects for matching to a given subject that untreated subject whose propensity score is closest to that of the treated subject (Austin, 2011).

Matching the 95 subjects who were assessed to be depressed based on the STDS-T cutoff value resulted in a sample of 190 subjects. The standardized mean difference was close to 0 for all covariates, indicating a successful match.

## Results

### Descriptive Statistics

Participants reported an average score of 3.22 on consistent contraceptive behavior in the past 3 months (1 = "not at all" to 6 = "very consistently") in wave 3, corresponding to slightly below-average consistent contraceptive behavior, with a high dispersion of scores (SD = 1.79). A total of 158 of 190 participants reported having used contraception within the last 3 months, 61 of them reported having used the birth control pill, 35 reported having used condoms, and 57 reported having used both. The remaining 4 reported having used other methods such as other hormonal drugs, natural contraception, or coitus interruptus. The mean value of the STDS-T sum score was below the cutoff value (*M* = 21.77, SD = 7.26). On average, an ambivalence value of 0.48 was found (compare Table 1).

**Table 1:** Descriptive statistics of total sample and subgroups with and without depression

	*N*	Minimum	Maximum	Mean	SD
STDS-T sum score	190	10.00	38.00	21.77	7.26
Subjects with depression	95	25.00	38.00	28.27	3.10
Subjects without depression	95	10.00	24.00	15.28	3.35
Ambivalence (without negatives)	181	0	3	0.48	0.65
Subjects with depression	91	0	3	0.51	0.69
Subjects without depression	90	0	3	0.46	0.60
Consistency of contraceptive behavior	190	1	6	4.79	1.87
Subjects with depression	95	1	6	4.43	2.03
Subjects without depression	95	1	6	5.15	1.64

*STDS-T* State-Trait Depression Scale Trait Version

Depressed subjects had a smaller mean score of 4.43 on the six-point Likert scale of consistent contraceptive behavior than subjects without depression (*M* = 5.15). This difference was statistically significant, *t*(188) = − 1.95, *p* < 0.01. As expected, the mean of the STDS-T sum score in the subsample with depression is above the cutoff value at 28.27 (SD = 3.10).

In the depressed subsample, the mean ambivalence is slightly higher at 0.51 as compared to the non-depressed subsample with a mean ambivalence score of 0.46. Since this difference was not statistically significant (*t*(179) = 0.52, *p* = 0.619), we exploratively examined whether the ideal and realistic numbers of children were significantly lower in the depressed subsample using a one-tailed T-test. We found that subjects in the depressed subsample showed a significantly lower realistic number of children (*t*(188) = − 1.35, *p* = 0.029) and a marginally significantly lower ideal number of children (*t*(188) = 1.91, *p* = 0.089).

### Mediation Analysis

**Fig. 1:**
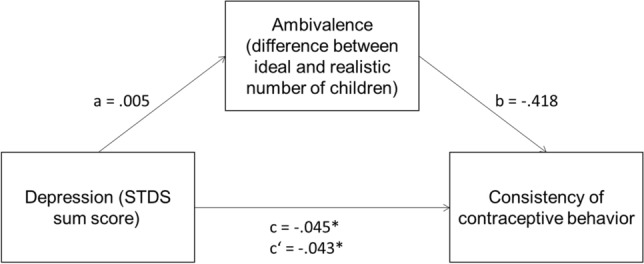
Results of mediation analysis for subjects without children with propensity score matching. *c*’ = relationship of depression and consistent contraceptive behavior after adding the mediator. *p < .05

Figure 1 presents the results of this mediation analysis. The association between the STDS-T sum score (wave 2) and consistency of contraceptive behavior in the past 3 months (wave 3) was significant, *c* = − 0.045, *p* = 0.02. This association was not affected by the inclusion of ambivalence in the model as a mediator *c*’ = − 0.043, *p* = 0.04. The relationship between depression and consistent contraceptive behavior was not mediated by ambivalence, indirect effect *ab* = − 0.0015, 95%-CI[− 0.0081, 0.0031]. The STDS-T sum score and ambivalence (the difference in ideal and realistic number of children) were not significantly correlated, *a* = 0.005, *p* = 0.49. Also, the association between ambivalence (wave 2) and consistency of contraceptive behavior in the last 3 months (wave 3) failed to reach significance (*b* = 0.418, *p* = 0.11).

**Fig. 2:**
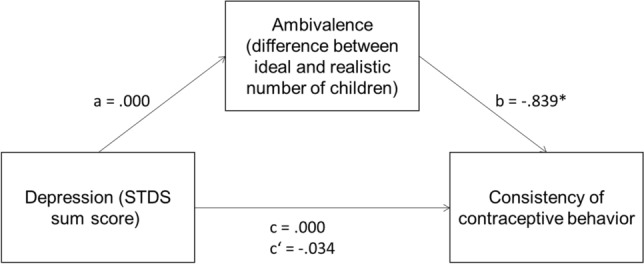
Results of mediation analysis, male subsample. *c*’ = relationship of depression and consistent contraceptive behavior after adding the mediator. *p < .05

When men and women were explored independently, the results were somewhat different. For men (see Fig. 2), the association between the STDS-T sum score and consistency of contraceptive behavior in the past 3 months was not significant, *c* = 0.000, *p* = 0.98, but ambivalence and consistency of contraceptive behavior in the last 3 months were significantly correlated *d*, *b* = − 0.839, *p* = 0.02.

**Fig. 3:**
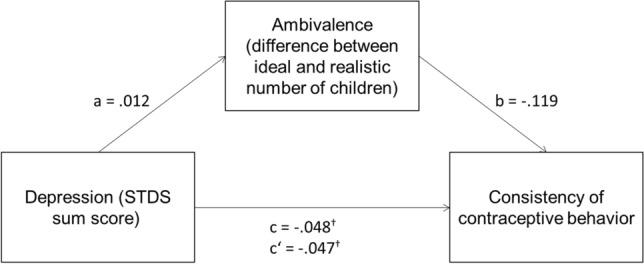
Results of mediation analysis, female subsample. *c*’ = relationship of depression and consistent contraceptive behavior after adding the mediator. *p < .05; ^†^*p* < .10

For women (see Fig. 3), the association between the STDS-T sum score and consistency of contraceptive behavior in the past 3 months only reached marginal significance (*c* = 0.048, *p* = 0.07, *c*’ = 0.045, *p* = 0.09), probably due to the smaller sample when men were not included. The other two pathways were not significant for women (*a* = 0.012, *p* = 0.12; *b* = − 0.119, *p* = 0.76).

In our mediation models, *R*^2^ was 0.05 for the entire sample, 0.11 for the male subsample, and 0.04 for the female subsample.

## Discussion

The aims of the present study were to examine the association between depression and inconsistent contraception that regularly has been found in previous research and to analyze the possible mediating role of ambivalence on it. In the mediation analysis, the association between the STDS-T sum score and consistency of the contraceptive behavior was indeed significantly negative (Hypothesis 1). However, ambivalence (included as a mediator) was not correlated with the STDS-T sum score. Thus, the results did not support the second hypothesis. For men, ambivalence and consistency of the contraceptive behavior were significantly and negatively correlated, as such partly supporting the third hypothesis. Finally, whereas we found no evidence for a mediation or partial mediation of the correlation between depression and consistency of the contraceptive behavior, for men ambivalence independently contributed to the consistency of contraceptive behavior.

### Hypothesis 1

STDS-T sum score and consistent contraception in the last 3 months were significantly correlated in our study. This finding is consistent with previous research. For example, a number of studies that explicitly examined the association of depression and consistent contraceptive behavior found consistent although small correlations (Hall et al., 2012, 2013; Kosunen et al., 2003; Lehrer et al., 2006; Nelson et al., 2017a, 2017b). All of these listed studies examined American samples. In contrast, the present study is based on a German-speaking sample. Note that in Germany access to contraceptives is ubiquitous (Helfferich, 2013) and, at most, only 5% of women have not used contraception in the past, despite sexual contact. In addition, men were also included in the present analysis, however, for them STDS-T sum score and consistency of contraception were not significantly correlated. Since many unintended pregnancies occur in Germany as well, it nonetheless is necessary to shed light on the causes of these pregnancies and thus on contraceptive behavior in further research.

Since the data of the present study originate from a panel study, it must also be taken into account that a drop-out of severely depressed individuals may have occurred from the first to the second and also from the second to the third wave. Furthermore, only individuals living in their own household were interviewed, those staying in clinics or residing in institutions were also not included.

### Hypothesis 2

In the mediation analysis, no significant relationship was found between depression and the ambivalence of fertility desires and expectations. The hypothesis can therefore not be confirmed with the available data.

Thus, the present work does not replicate the finding from the study by Patel et al. (2015), which found depression to be a significant predictor with a high coefficient for ambivalent pregnancy motivations. This may be due to several reasons. In the study by Patel et al., American women were examined. One possibility could be that the correlation cannot be transferred to German women and men. Since to the actual state of research, only the mentioned study has investigated this correlation at all, so more research is needed here. Another difference is the operationalization of the ambivalence concept. Here, the negative cognitive triad and in particular the negative future expectations were taken into account, which have a negative impact on fertility desires (Margraf et al., 2022), and probably also on fertility expectations. Fertility expectations were operationalized by the realistic number of children and fertility desires by the ideal number of children. Operationalizing ambivalence as the difference between these two numbers represents an extension of the concept of ambivalence. Thus, the operationalization could also be a reason for the nonsignificant relationship. Our exploratory results of our data suggest that subjects with depressive symptoms show both a lower realistic and lower ideal number of children. If depressive symptoms equally affect fertility desires and fertility expectations, it can be assumed that the difference between the two is not larger for individuals with depressive symptoms than for those without.

### Hypothesis 3

The third hypothesis, assuming a negative relationship between ambivalence and consistency of contraceptive behavior, is supported by the data, but only for men. These findings replicate the results of the only study that involved men in their sample (Higgins et al., 2012). As such, despite the different operationalization of the ambivalence concept explained in the discussion of Hypothesis 2, the present work partly replicates the current state of research regarding the relationship between ambivalence in pregnancy intention and consistent contraception. This may be taken as an indication of convergent validity and suggests that the transfer of the ambivalence concept from pregnancy motivation to pregnancy intention as well as the formation of ambivalence as the difference between the ideal and realistic number of children may be considered as valid. The results of the present analysis extend the actual state of research by indicating that the studied relationship can be applied to German-speaking, sexually active men.

For men, the B pathway is the main meaningful significant pathway. This emphasizes the importance of the ambivalence concept for consistent contraceptive behavior. Given that unintended pregnancies are also quite common in Germany, the results suggest that it is highly relevant to investigate this relationship in more detail in order to be able to implement adequate interventions to counter the negative consequences of unintended pregnancies.

### Hypothesis 4

Ambivalence does not mediate the relationship between depression and consistency of contraceptive behavior. Given the large sample, the power of the present analysis is sufficient to interpret this as a lack of mediation (Fritz & Mackinnon, 2007). To our knowledge, ambivalence has not been investigated as an influencing factor on the relationship between depression and consistent contraceptive behavior. As already explained above, no significant positive association was found between depression and ambivalence. It can be assumed that this lack of association is partly responsible for the missing mediation effect. Note, however, that one previous study (Patel et al., 2015) also examined this association and was able to identify depression as a significant predictor of ambivalence. Different operationalization of ambivalence might also have led to different results.

The aim of the present study was to investigate the relationship between depression and consistent contraceptive behavior in more detail and to consider ambivalence as a possible mediating influencing factor. While ambivalence conceptualized as a mediator did not exert any influence, we found a direct effect of ambivalence on consistent contraceptive behavior for men.

### Limitations, Strengths, and Implications

First, it is important to note that pairfam is based on data from Germany only, as such from a Western society. As such, our results may not be generalizable to people from other nations.

Furthermore, the operationalization of consistent contraceptive behavior could clearly be improved. For example, the retrospective self-report regarding the last 3 months may be subject to memory bias. These self-report methods have limitations due to recall, social desirability, and selection biases, as respondents tend to overestimate adherence (Nelson et al., 2017a, 2017b). An objective measurement of contraceptive behavior is nevertheless difficult to implement in practice. In addition, it was not possible to ensure that no women with premature menopause were included in the sample.

The variable of consistent contraceptive behavior was also merged from two different variables. Accordingly, it is important to note when interpreting the descriptive statistics that the center of the scale has shifted by one point and there is no longer a middle point on the six-point Likert-type scale like before when the subjects answered the question. In addition, difference scores are less reliable given that two standard errors are added up.

Another issue is the detection of the presence of depression using the STDS-T questionnaire. This questionnaire was developed for non-clinical samples. Even though studies show that the questionnaire is a valid and reliable diagnostic tool (Guillot Valdés et al., 2020; Krohne et al., 2002; Lehr et al., 2008), more common survey instruments would lead to a better comparability of the studies.

Finally, the pairfam study is designed as a panel study. In a long-term study, both accessibility and reliability must be guaranteed in order to achieve regular participation. It can be assumed that especially people with depressive symptoms have difficulties in maintaining regular participation. To address this problem, data from the second and third wave were used for the analysis. Given that the first wave could not be used due to a lack of survey instruments, some dropouts may have already occurred. In addition, only persons currently living in a household in Germany were included. Individuals being treated in clinics or in institutions were thus excluded, who possibly would have been highly relevant for the present work.

However, the present study also had some strengths. Due to the very elaborate randomized recruitment from communities all over Germany and from different cohorts, the results can be generalized to a certain extent to sexually active, German-speaking adults who live in their own households. Especially the inclusion of men in the sample should be emphasized positively at this point, since the previous studies mainly referred to women only. This allows insights that can be applied to both genders. Furthermore, the concept of ambivalence, which has so far only been applied in the context of pregnancy motivation, was transferred to the concepts of fertility expectations and desires. The data here speak for a successful application of the ambivalence concept in this new research area.

This study addressed a very important point by examining the relationship between depressive symptoms and consistency of contraceptive behavior. The connection between these two factors has been shown many times and could now be replicated in a German sample; however, the effect is small. The present study thus lays an important foundation for further research in transnational samples.

### Future Research

The limitations of the study, as well as the general current state of research, provide some impetus for future research.

Our results suggest a successful, valid transfer of the concept of ambivalence from motivation to intention and behavior. Thus, ambivalence appears to be influential at various stages with regard to sexual behavior and may be transferable to other core concepts in this domain as well as in other domains. Accordingly, further research on the ambivalence construct should be conducted. At least in German samples, this concept seems to be important to better understand contraceptive behavior.

Consistent contraceptive behavior can be operationalized in very different ways. In the present study, a retrospective self-report was used. However, in order to understand inconsistent contraceptive behavior better and without bias, it would be useful in future studies to record consistent contraceptive behavior at shorter intervals over several time points in a longitudinal study.

Since ambivalence does not seem to be a mediator in the connection between depression and inconsistent contraceptive behavior, future research should also aim at finding other mediators. Aspects such as higher rates of intimate partner violence or higher frequency of somatic side effects of hormonal contraception have been discussed in the literature (Catalao et al., 2023). Also, depression may be associated with reductions in the cognitive functioning essential to remembering and following through with adherence to oral contraceptive pills (DiMatteo et al., 2000). In order to prevent unplanned pregnancies in individuals with depression, more research is needed to understand the underlying mechanisms.

## Data Availability

Publicly available datasets were analyzed in this study. This data can be found at the German Family Panel (pairfam). GESIS Data Archive, Cologne. ZA5678 Data file Version 12.0.0, https://doi.org/10.4232/pairfam.5678.12.0.0.

## Footnotes

1 This paper uses data from the Relationship and Family Panel pairfam, which is led by Josef Brüderl, Sonja Drobnič, Karsten Hank, Franz Neyer, and Sabine Walper. The study is funded as a long-term project by the German Research Foundation (DFG). The principal investigators of the pairfam project are Josef Brüderl (Mannheim), Johannes Huinink (Bremen), Bernhard Nauck (Chemnitz), and Sabine Walper (Munich).
